# Supplementary figures and images for: Thermodynamic constraints on the assembly and diversity of microbial ecosystems are different near to and far from equilibrium
Source: PLoS Comput Biol. 2021 Dec 3;17(12):e1009643. doi: 10.1371/journal.pcbi.1009643 (PMC8673627; doi:10.1371/journal.pcbi.1009643)

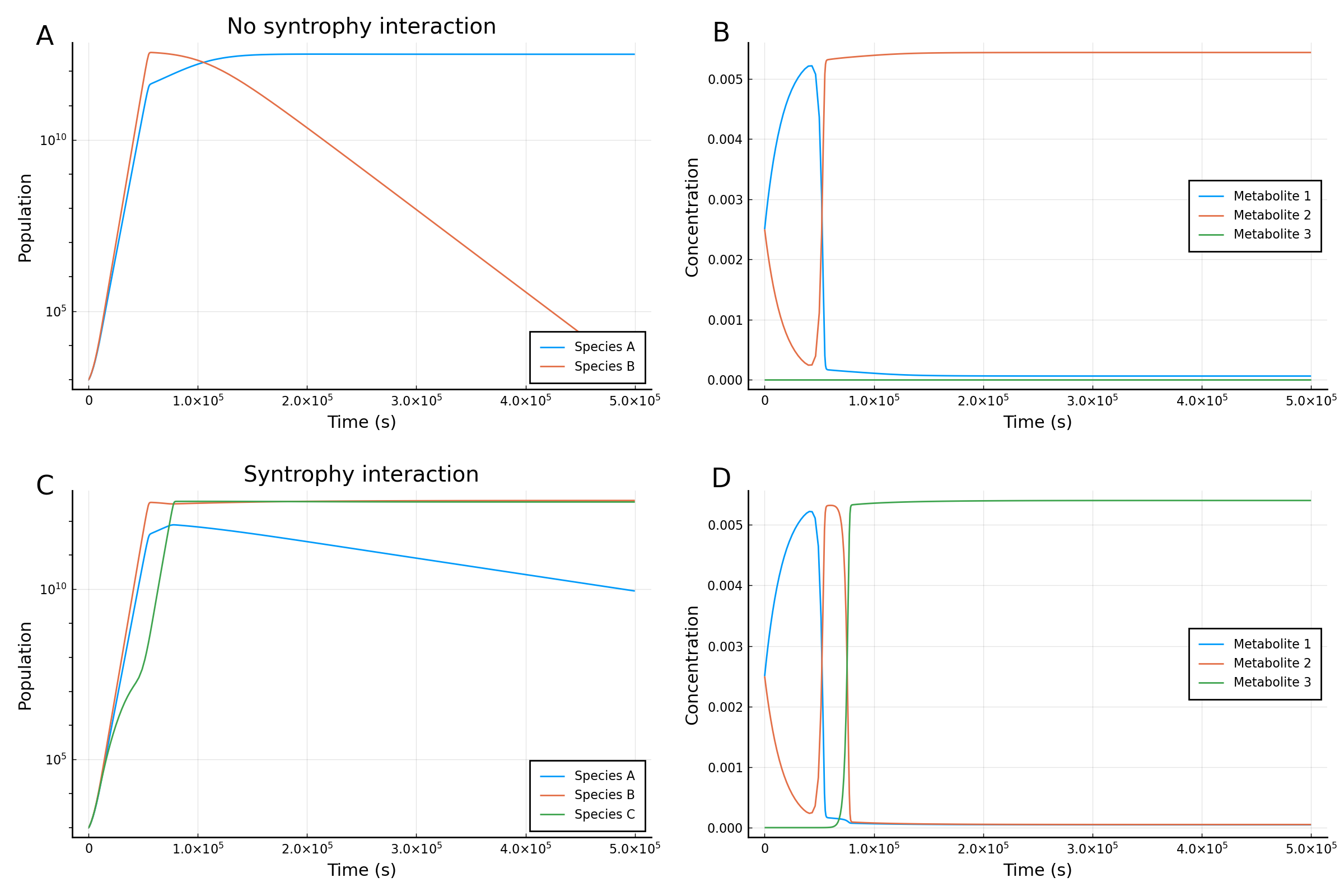

Supplement: S1 Fig — Our system here consists of three metabolites (only the first of which is supplied) and three species. The species A and B both break down metabolite 1 to produce metabolite 2, and species C breaks down metabolite 2 to produce metabolite 3. Species B generates more ATP per mole of reaction than A does. A: When species A and species B are grown together species A initially grows faster due to its greater ATP yield. However, as steady state is approached species A dies off to be replaced by species B. B: Species A dying off occurs due to the build of metabolite 2, which inhibits species A more due to it being closer to thermodynamic equilibrium. C: When species C is included, species A now survives to steady state (along with species C) instead of species B. D: The concentration of metabolite 2 is reduced due to consumption by species C, reducing the thermodynamic inhibition of species A. We term this a syntrophy interaction. (TIF) [file pcbi.1009643.s002.tif]

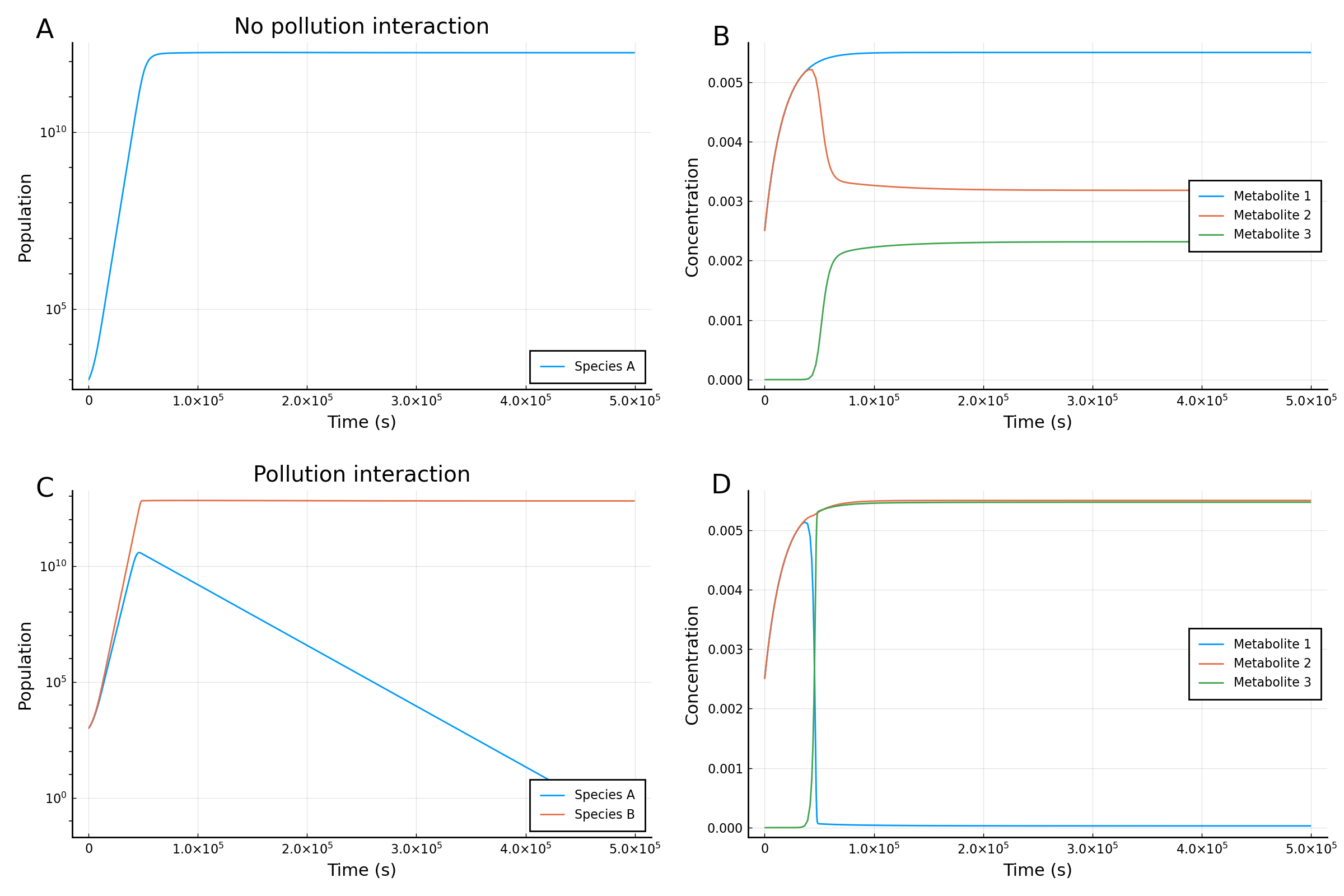

Supplement: S2 Fig — Our system here consists of three metabolites (the first two of which are supplied) and two species. Species A breaks down metabolite 2 to produce metabolite 3, and species B breaks down metabolite 1 to produce metabolite 3. A: When species A is grown on its own it reaches a steady state population. B: As species A only breaks down metabolite 2, both metabolite 1 and 3 accumulate. C: When species B is added to the system species A is driven to extinction. D: In this case, greater accumulation of metabolite 3 occurs as species B breaks down metabolite 1 into it. Species A therefore experiences a greater level of thermodynamic inhibition. As species A and B do not share a substrate this competitive exclusion occurs purely via waste products, we therefore term this a pollution interaction. (TIF) [file pcbi.1009643.s003.tif]

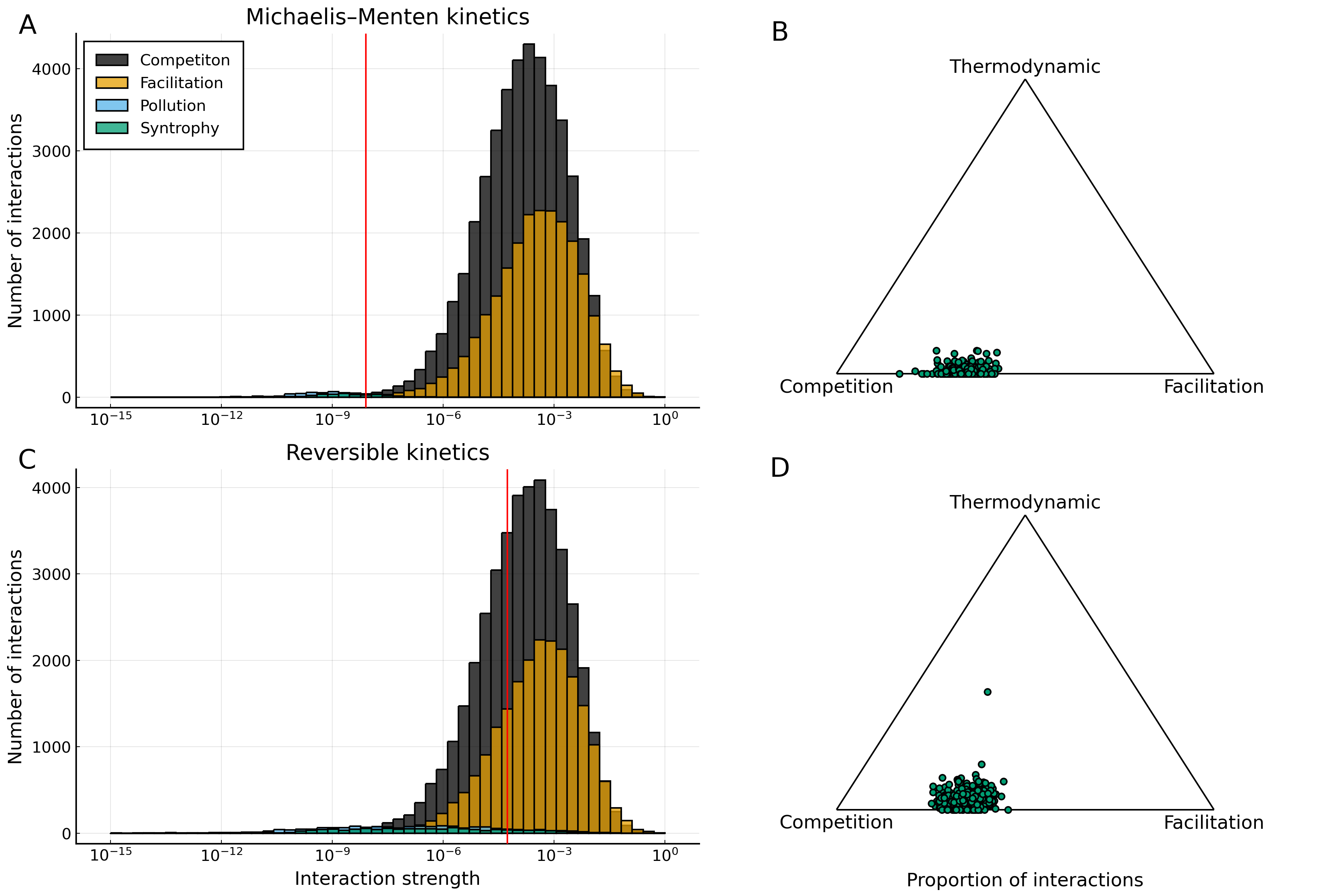

Supplement: S3 Fig — Identical plot to Fig 5 but for the high energy supply case (1.5 × 10^7^ J mol−1). (TIF) [file pcbi.1009643.s004.tif]
