# Supplementary material for: Thermodynamic constraints on the assembly and diversity of microbial ecosystems are different near to and far from equilibrium
Source: PLoS Comput Biol. 2021 Dec 3;17(12):e1009643. doi: 10.1371/journal.pcbi.1009643 (PMC8673627; doi:10.1371/journal.pcbi.1009643)
Supplement: Attachment — Submitted filename: Review-Endres-2021.pdf [file pcbi.1009643.s005.pdf]

In their manuscript "Thermodynamic constraints on the assembly and diversity of microbial ecosystems are different near to and far from equilibrium", Cook *et al* seek to combine ecological consumer resource models, recent advances on models of cell growth and proteome allocation pioneered, and basic thermodynamic models of regulation into a coherent picture of microbial ecosystems. This is largely done through simple numerical simulations. While the topics are interesting and the investigators are well qualified to carry out this research, overall, I found the manuscript quite confusing and the central result suggested in the title to be an overstatement of their simulation results given the many assumptions. Also, I think it would be helpful if the author dialogued more with the existing literature.

I now provide some more specific comments.

1. Throughout the manuscript, their seems to be a general confusion/lack of clarity about the difference between anabolic and catabolic processes and how they are coupled. The variable *a* of course refers to anabolic processes whereas reproduction of proteins is of course catabolic. The coupling comes from the very strong assumption that energy is used only for translation. This is far from obvious and given the weak effect size in Figure 4, I am very unconvinced of the main result.

2. There are also a number of arbitrary assumptions that make it hard to assess whether to take the effect of thermodynamics seriously. Just to a give few examples:

- "Although the fraction of ribosomes bound (f_b) would be expected to vary with the cells internal energy concentration, as a simplifying assumption we take it to be fixed at a value of 0.7 "
- Eq. 7 for main text and choice of Phi_q for optimum ribosome fraction
- The fact that one ignored anything is a processes with large free energy differences and more than one product

The robustness of the result needs to be checked to show it is robust to relaxing/changing all these assumptions (which should be stated in a table and made more explicit instead of being interspersed through out main text/SI

3. The authors have to dialogue with another manuscript that combined consumer resource models and proteome allocation constraints:

Pacciani-Mori, L., Suweis, S., Maritan, A. *et al.* Constrained proteome allocation affects coexistence in models of competitive microbial communities. *ISME J* **15,** 1458–1477 (2021). https://doi.org/10.1038/s41396-020-00863-0

This model has some very interesting ideas and results about dynamics which seem to be much more robust and dramatic then the simulations here (be it in a narrow set of

input resource fluxes). I am not sure how to reconcile the results here from there. More generally, a comparison of assumptions and how they affect results would be very important for me to really understand what the thermodynamic framework adds.

4. The authors should also dialogue more explicitly with Ref 20 which also showed that the primary driver of diversity is energy availability due to the number of intermediate substrates. What new do I learn from incorporating all these additional assumptions? It was also shown that ordinary consumer resource models can also produce modularity (see Marsland 2020 Scientific Reports). It would be helpful to distinguish the results of this new model from those in these papers.

4. The two-step interaction calculation is very reminiscent of a cavity calculation. Perhaps, since everything is random one can figure out average effect size by considering invasion growth rates and changes in steady-state concentrations (see for example Advani et al J. Stat Mech 2018; Cui et al PRL 2020). It would be interesting to better understand the relationship between these methods and whether one is in effect calculating the cavity susceptibilities.
