# Supplementary material for: Thermodynamic constraints on the assembly and diversity of microbial ecosystems are different near to and far from equilibrium
Source: PLoS Comput Biol. 2021 Dec 3;17(12):e1009643. doi: 10.1371/journal.pcbi.1009643 (PMC8673627; doi:10.1371/journal.pcbi.1009643)
Supplement: Attachment — Submitted filename: Response_to_reviewers__PCOMPBIOL_D_21_01016_.pdf [file pcbi.1009643.s006.pdf]

Responses to the Reviewers

Comments made by Reviewers are in **black**, whereas our responses are in **blue**.

All revisions that we have made to the manuscript mentioned or referenced below are also highlighted in the revised manuscript.

Reviewer #1

The authors construct an artificial model of microbial communities based on random reaction networks for each modeled species governed by thermodynamics, a limited protein pool and either irreversible or reversible reaction kinetics. The inclusion of a protein budget (so far only utilized on an individual species level) and thermodynamics in a community-scale model is novel and has to my knowledge not been attempted yet. They then proceed to investigate community dynamics and interactions through simulations of the model testing different scenarios (high/low energy substrates and reversible/irreversible kinetics). While an approach like this to some extent always begs the question of how representative such an artificial model is of real microbial communities, I find its application very interesting and useful in terms of assessing different modes of how thermodynamics might influence microbial community composition, interactions and dynamics (something that would by very hard/impossible to do based on real microbial communities due to a lack of thermodynamic information and other necessary state variables).

Minor revisions

---

1. I am sorry if I missed it, but does your model accurately capture that reactions that operate close to equilibrium need very high enzyme levels (thus resulting in high protein expression costs affecting growth rate) to compensate for the low thermodynamic driving force (see [1]). As growth rate only depends on ribosome fraction and energy availability in your model (and not also explicitly on production of "biomass" precursors from substrates at specific rates), I would assume that reactions close to equilibrium would have very low metabolic rates and the cells relying mainly on reactions that are further away from equilibrium to generate energy, while in reality cells would have to maintain certain reaction rates to facilitate biomass precursor production to achieve a certain growth rate? Could you please elaborate on how this might affect the main outcomes of the study?

   Our model only explicitly includes catabolic reactions, with all anabolic reactions being abstracted by the energy concentration parameter a. The reviewer is correct to note that this results in cells relying mainly on reactions that are further from equilibrium to generate energy, with reactions close to equilibrium having low metabolic rates relative to the proteome dedicated to them. Thermodynamic (and kinetic) bottlenecks often do occur for anabolic reactions in real cells, necessitating significantly increased metabolic protein investment to sustain growth. Because anabolic reactions are not explicitly included in our model these bottlenecks cannot be modelled directly, however, they can be approximately captured by increasing the the average metabolic enzyme mass n_P. This captures the increased anabolic protein cost due to bottlenecks, resulting in a reduction in the number of catabolic proteins (Eq 9), for a given metabolic proteome fraction (ϕ_P). We expect the results of our paper to be unchanged when n_P is varied, though with decreased final diversities observed when it is increased, and species with lower values of n_P being favoured. It is also worth noting that we considered a (catabolic) reaction network without any thermodynamic bottlenecks, i.e. the free-energy change of every step downwards in our metabolite hierarchy (see Fig 1B) was equal. Thus, a complete (and functional) metabolic network could emerge without any species possessing a reaction close to thermodynamic equilibrium. Reactions close to equilibrium emerge due to particular species transducing

a sufficiently large fraction of the reaction free-energy (as ATP), rather than species using inherently bottlenecked reactions. If inherently low free-energy change reactions were introduced to the overall network, then one or more species in the community would have to make a substantial proteome investment in these reactions in order for the community to develop fully.

We have now clarified the role of metabolic and catabolic processes in our model (lines 64–65) and discussion (lines 471–474), where we compare our results with those of [1]. This issue is also touched upon in our new supplementary text section S9 that deals with the assumptions underlying our model, where we establish the robustness of our results to an increase in the average metabolic enzyme mass (n_P).

2. I find the manuscript is lacking details on the occurring interactions and the presentation of the results is primarily from a high level perspective. For example, l. 422-424 in the Discussion mentions

   > ... with competition occurring primarily within functional groups, facilitation and syntrophy occurring primarily between functional groups, and pollution occurring both within and between neighbouring functional groups.

   but these results are not presented?

   In our model species are assigned to functional groups based on the substrate of their most expressed reaction. From this it follows that competition for substrates occurs primarily within functional groups. The other three results stem from similar logic, but we now acknowledge that there are unlikely to be obvious to anyone not intimately familiar with the details of our approach. To address this we have added a section (S7) to the supplementary text addressing this result, which includes two new figures consisting of heat-maps of the strength and frequency of the different interaction types across our simulations. This section is now referred to in the Discussion (lines 519–520).

3. In line with the previous comment, I think it could be worthwhile highlighting concrete examples of how pollution and syntrophy arises in the simulations (maybe in the Supplements)?

   We agree, that concrete examples of how pollution and syntrophy interactions arise would help readers to understand our results. We have therefore made new plots showing examples of syntrophy and pollution interactions occurring in systems with a small number of species and metabolites. These are included as supplementary figures (Figs S1&S2) and reference is made to them in the main text (lines 207–209).

4. Niebel, Leopold & Heinemann [2] have reported the existence of an upper rate limit on Gibbs energy dissipation (organism specific). I would be interested to hear your take on this result in the light of your simulations. Given the existence of such a limit, would one expect maybe an increased proportion of thermodynamic interaction than the simulations currently result in? Maybe you could add that to or after the paragraph at l. 378-388?

   In Niebel *et al.* a maximum free energy dissipation rate is observed. At a certain point the microbe they study shifts to using a reaction that dissipates less free energy relative to the free energy retained for growth, resulting in a maximum free energy dissipation rate. Cells cannot adapt their metabolism in such an manner in our model, but we still expect the emergence of a maximum possible rate of free-energy dissipation through a different mechanism. Due to the finite proteome and bounded kinetic parameters in our model, a maximum metabolic flux is implied. This then implies that for a given substrate free-energy, a maximum rate of free-energy dissipation exists. However, due to competition

between strains this maximum rate isn't achieved and species settle to a lower average rate of entropy production (n.b. the spikes in Fig 3B). This maximum rate emerges from proteomic rather than thermodynamic constraints, suggesting a possible link between the results obtained by Niebel *et al.* and explanations for the existence of overflow metabolism based on proteomic constraints [3]. If Niebel *et al.*'s result is taken as given, and there is indeed a maximum (species specific) rate at which entropy can be removed from a cell, then we would indeed expect thermodynamic interactions to play a larger role in real ecosystems. This is because this would represent a further favouring of thermodynamically efficient reactions, as in addition the benefit of greater ATP generation rates, rates of entropy production would be lower. However, this will only be a strong effect for species that produce entropy at a rate close to the maximum removal rate, which probably only occurs during phases of rapid growth.

To address all this we have added a paragraph (lines 476–497) to the Discussion session on this relevant paper as suggested.

5. l. 23, Microbes experience many more physical constraints than just the two constraints mentioned, see [4].

   To avoid giving a misleading impression, we have now changed this section (lines 23–25) to mention that more physical constraints do in fact exist and to refer the interested reader to this useful reference.

6. Are all metabolites consumed/secreted or are there internal-only metabolites also? Based on l. 82, it sounds like all metabolites are accessible to all species at all times? That would be very far from reality? Beyond maintaining concentration gradients, cellular membranes will prohibit many metabolites from freely diffusing into the environment? Since all metabolites are also external and can thus facilitate thermodynamic inhibition through products? Is that realistic?

   The reviewer is correct to note that in our model all metabolites are accessible at all times. This reduces computational cost, allowing us to track 25 metabolites in total without also having to track an additional 25 metabolites for every species included in our model. This obviously represents a significant simplification of reality, but is a common assumption in microbial consumer-resource modelling (e.g. [5]), where a focus on the community context necessitates less detail for individual species. However, including internal metabolites explicitly in our model would cause either no change in or an increase in the strength of the thermodynamic effects. In terms of cell membranes impeding diffusion we expect that this would lead to increased local product buildup; this would decrease thermodynamic interactions between cells, but would increase the impact of thermodynamic inhibition on individual cells. Alternatively, by maintaining concentration gradients (through uptake and excretion) cells can maintain metabolic reactions away from equilibrium, this is particularly important for anabolic reactions. However, for the catabolic reactions that we consider in this manuscript the free energy cost of maintaining a particular concentration gradient will always, due to the 2nd law of thermodynamics, be greater than the reaction free-energy contributed by the gradient. It is therefore appropriate to regard our results as a lower limit on the strength of thermodynamic inhibition on microbial communities.

   This assumption is already mentioned in the model section (as the reviewer notes), but we now discuss the implications of making this assumption in the Discussion (lines 458–469). This assumption is also addressed (among others) in a new supplementary text section (S9).

7. The authors use Michaelis–Menten kinetics synonymously for irreversible kinetics though a

reversible Michael-Menten rate equations exists? Maybe clarify that you specifically mean the original rate law developed by Michaelis and Menten?

We agree that this is potentially confusing and have clarified this in the manuscript in the following ways: (1) changed our first mention (line 101) of Michaelis–Menten kinetics to "irreversible Michaelis–Menten kinetics", (2) changed the definition of "Michaelis–Menten" kinetics in the Simulations section (lines 236–237) to clarify that we are considering the irreversible case and (3) similarly clarified this in the relevant section (S1) of the supplementary text.

8. eq. 1 and accompanying text, you use α here to reference metabolites though for the rest of the manuscript you have reserved β for metabolites and α for reactions.

   We agree that using subscripts consistently here would improve the clarity of the text, and so we have made the suggested change to Eq 1 and the accompanying text (lines 67 & 71–74).

9. l. 198, how connected and functional are those randomly chosen metabolite networks?

   The overall catabolic network in our model is fully connected, with the lowest free-energy metabolite always being reachable from initially supplied metabolite. Individual species are then assigned a small (1-5 reactions) subset of this large (47 reaction) set. These subsets can be connected, but due to the low number of reactions they do not span much of the metabolite space. As the microbial community assembles its shared reaction network will tend to increase its connectivity, with a fully connected (catabolic) network (i.e. every metabolite being reachable from metabolite 1) being achievable when free-energy supply is high (see Fig 4B). As we consider purely catabolic networks even incomplete networks can be functional (i.e. able to support growth) provided there is a reaction breaking down the initially supplied substrate. However, incomplete networks can support fewer strains (see Figs 4A&B). We have now added a discussion of the completeness of the various types of network when they are first introduced to the manuscript (lines 142–147).

10. Fig 2b, why do all surviving species reach the same ribosome fraction? Is that expected?

    Given the setup of our model this is an expected result because all species possess an equal rate of biomass loss d; thus at steady state all species must possess equal growth rates, i.e. $\lambda = d$. If saturation constants ($\gamma_{\frac{1}{2}}$, $\Omega_{\frac{1}{2}}$) are not fixed across species, different ribosome fractions supporting the same growth rate (at steady-state) can feasibly be observed, with higher energy concentrations (a) compensating for lower ribosome fractions. However, when saturation constants are fixed across strains no strain can have a higher energy concentration and a lower ribosome fraction. This is because the ribosome fraction is determined by the energy concentration (see Eq 7 main text). We now explain this result when it first occurs (lines 256–259), and justify our fixing the two saturation constants across species in the Discussion (lines 529–535)

11. Reaction efficiency is not clearly defined I think (I am sorry if I overlooked it)

    We apologize for this oversight. We have now added a new supplementary material section S4 that defines reaction efficiency.

12. l. 301-302, low free energy should be recalcitrant and high free energy should be labile, or?

    The high and low free energy cases were unfortunately mislabelled here, we have now corrected this (lines 335–337).

13. It is great that you provided all your code on GitHub, but could you maybe highlight in your READMEs main entry points (like files that produces the figures?)

    The README has now been altered to clearly indicate the scripts which generate the simulation data and the various figures.

Typos and grammar

---

14. Last sentence in abstract, delete "a" in "of a real world"

    We have now corrected this typo. Thanks for pointing it out.

15. MacArthur consumer-resource resource model -> MacArthur consumer-resource model (?)

    We have now corrected this typo. Thanks for pointing it out.

16. While your writing is very good, please check comma usage of introductory clauses to separate them from main clauses, e.g., l. 158, "As η changes, the reaction ...", l. 246, "For all regimes, ...", etc. throughout the manuscript

    We thank the reviewer for pointing out this area for improvement. We have now checked that commas are used correctly in all introductory clauses beginning with 'From', 'Here', 'When' 'If', 'As', 'For', 'Although', 'Thus', 'Hence', or 'In'. In doing this we have added a number of extra commas (lines 50, 119, 173, 176, 181, 183, 193, 226, 235, 280, 306, 343, 499 & 507).

17. l. 189 the the

    We have now corrected this typo. Thanks for pointing it out.

18. l. 343-345, the sentence does not make sense

    Looking at this sentence we agree that the wording is unnecessarily convoluted. In order to make our argument clearer we have now split this sentence into two separate sentences (lines 378–382).

Reviewer #2

In their manuscript "Thermodynamic constraints on the assembly and diversity of microbial ecosystems are different near to and far from equilibrium", Cook *et al.* seek to combine ecological consumer resource models, recent advances on models of cell growth and proteome allocation pioneered, and basic thermodynamic models of regulation into a coherent picture of microbial ecosystems. This is largely done through simple numerical simulations. While the topics are interesting and the investigators are well qualified to carry out this research, overall, I found the manuscript quite confusing and the central result suggested in the title to be an overstatement of their simulation results given the many assumptions. Also, I think it would be helpful if the author dialogued more with the existing literature.

1. Throughout the manuscript, their seems to be a general confusion/lack of clarity about the difference between anabolic and catabolic processes and how they are coupled. The variable a of course refers to anabolic processes whereas reproduction of proteins is of course catabolic. The coupling comes from the very strong assumption that energy is used only for translation. This is far from obvious and given the weak effect size in Figure 4, I am very unconvinced of the main result.

   Our model only explicitly models catabolic reactions, with anabolic processes being abstracted by the ribosome proteome fraction. This does indeed rest on the assumption that energy is only used in protein translation. Protein translation is known to be one of the

largest sinks of energy in microbial cells [6], this cost includes not just the cost of translational elongation but the cost of synthesising the necessary amino-acids. The cost per elongation step χ used in our model incorporates both costs. Some of the other processes that use cellular energy are growth associated, such as the costs associated with chromosome duplication, transcription, and obtaining precursors necessary for biomass growth. As our model is already relatively complex, we deemed it reasonable to ignore these details and only consider protein translation. There are also cellular uses of energy that are not directly connected to growth metabolism (especially, motility, sensing and signalling). In these cases, energy that could have been used for growth is instead used to gain a competitive or cooperative advantage. The advantage gained by investing energy into such mechanisms can only really be captured in more complex spatially explicit models (e.g. [7]). Given the lack of this spatial consideration in our model, we therefore chose to neglect the energy used in these processes. We have revised the manuscript to make clear which processes are anabolic and catabolic (lines 64–65) and to make clearer the assumption we are making about protein translation being the main consumer of cellular energy (lines 65–67). These assumptions are also addressed (along with the other assumptions underlying our model) in a new section (S9) of the supplementary text.

2. There are also a number of arbitrary assumptions that make it hard to assess whether to take the effect of thermodynamics seriously. Just to a give few examples:

   We agree with the reviewer that our assumptions could be more clearly set out. We have done this in a new section of the Supplementary text (S9), which includes a table (Tab ST1) summarising the assumptions underlying our model. We will now address each of the specific criticisms below.

   > "Although the fraction of ribosomes bound (f_b) would be expected to vary with the cells internal energy concentration, as a simplifying assumption we take it to be fixed at a value of 0.7"

   The value used here was taken from [8]. As mentioned this would be expected to vary both between species and as the state of the cell changes. However, our results are robust to changes in the value of f_b. Increasing (decreasing) f_b would lead to an increase (decrease) in the growth rate for a given internal energy concentration (a). Using a higher value for f_b would therefore lead to a (slightly) faster rate of population growth, and thus a faster rate of ecosystem development. This faster rate of ecosystem development would however entail a greater usage of free-energy, so we would expect the observed differences based on free-energy supply to persist. The robustness of our overall results to an increase in f_b is now demonstrated in Fig ST6.

   Eq. 7 for main text

   The form of Eq 7 was chosen to be saturating with respect to the internal energy concentration a. This was inspired by the work of Weiße *et al.* [9] where a similar saturating function was used (in a more complex mechanistic model) to capture shifting of ribosome fractions associated with the empirically observed growth laws [10]. As shown in Fig ST1 (in the supplementary text), our model can also recover these growth laws. Replacing this function with any other saturating function would not effect our results qualitatively. This is because a function that adapts more slowly will cause more pronounced shifts in the internal energy concentration, meaning that slower adaptation with respect to the internal energy concentration would be counteracted by more rapid shifts in the internal energy concentration with changing substrate availabilities. This assumption is now justified in Tab ST1.

   and choice of ϕ_Q

The maximum possible size of the ribosome fraction is given by $(1-\phi_Q)$, with the value of the 'housekeeping' proteome fraction (ϕ_Q) being taken from [11]. Varying ϕ_Q for all species simultaneously would have a very similar effect to that of varying f_b, discussed in the previous comment. Among real cells the size of the 'housekeeping' proteome fraction would be expected to vary. This would arise from cells making a greater than average protein investment in processes not directly related to growth or energy acquisition, such as motility and chemotaxis [7], or preventing protein misfolding at high temperatures [12]. Our model does not capture the impact of these processes so any strain with a higher 'housekeeping' fraction would be disadvantaged due to having less proteome to dedicate to growth or energy acquisition without obtaining any compensating benefit. The robustness of our overall results to an increase in ϕ_Q is now demonstrated in Fig ST6.

for optimum ribosome fraction

The ribosome fraction that is obtained for a particular energy concentration is determined by the relevant half-maximum constant ($\Omega_{\frac{1}{2}}$). For the proteome model to function sensibly this constant must be of a similar order of magnitude to the corresponding half-maximum constant for the translation rate $\gamma_{\frac{1}{2}}$ (see Eq 6). If $\gamma_{\frac{1}{2}}$ is substantially larger, then cells will end up with a large ribosome fraction that only operates at a small fraction of the maximum rate. Whereas if $\Omega_{\frac{1}{2}}$ is substantially larger, ribosomes will always operate at maximum speed but only a vanishingly small fraction of the proteome will be dedicated to them. Neither scenario is consistent with the empirically observed growth laws. The absolute size of the two constants determines the internal energy concentration necessary to achieve maximal growth, making use of smaller values would remove all proteomic constraints on growth. However, sustaining this growth rate would require a very large energy flux, which would be particularly hard to obtain with almost the entire (non-housekeeping) proteome allocated to ribosomes rather than metabolic protein. Thermodynamic limitations on growth rate would therefore remain important even in the small $\gamma_{\frac{1}{2}}$ limit. We specifically chose $\Omega_{\frac{1}{2}}$ to be slightly larger than $\gamma_{\frac{1}{2}}$ to ensure that translation saturates quicker than the ribosome fraction shifts, in order to avoid the situation where species frequently fail to grow due to possessing a large number of inefficient ribosomes. We now demonstrate the robustness of our overall results to various changes in the values of $\Omega_{\frac{1}{2}}$ and $\gamma_{\frac{1}{2}}$ in the new supplementary text figure Fig ST5.

The fact that one ignored anything is a processes with large free energy differences and more than one product

We only considered cases where multiple products were generated for the sake of simplicity. However, the reaction quotient Q is proportional to the power of the product stoichiometry and the inverse power of the substrate stoichiometry. This means that we have considered thermodynamic effects in the limit where they are most weakly affected by changes in metabolite concentrations. The reason that anabolic processes with large free energy differences (specifically protein synthesis) are not treated explicitly in our model, is that when free energy differences are large the difference between reversible and irreversible kinetics disappears. This means that including a full reversible model of protein synthesis would add minimal insight at a cost of significant increased complexity. The choice to only consider single-reactant single-product reactions is now justified in a new subsection of the supplementary text (S9.3)

The robustness of the result needs to be checked to show it is robust to relaxing/changing all these assumptions (which should be stated in a table and made more explicit instead of being interspersed through out main text/SI

We agree that it is important to demonstrate the robustness of our results. To address this,

we have added plots (Figs ST5-6) to the new SI section (S9) demonstrating the robustness of our results to changes in various model parameters. Specifically, we demonstrate the robustness of our results to an increase in f_b, an increase of the 'house-keeping' fraction ϕ_Q, an increase of the relative size of the translation half-maximum constant $\gamma_{\frac{1}{2}}$ compared to the ribosome half-maximum constant $\Omega_{\frac{1}{2}}$, and both an increase and a decrease of the size of both half-maximum constants.

3. The authors have to dialogue with another manuscript that combined consumer resource models and proteome allocation constraints [13]. This model has some very interesting ideas and results about dynamics which seem to be much more robust and dramatic then the simulations here (be it in a narrow set of input resource fluxes). I am not sure how to reconcile the results here from there.

   Pacciani-Mori *et al.* [13] combine a proteome allocation model with a consumer-resource model. The proteome allocation model they used is broadly similar to ours (fixed ϕ_Q, etc), though proteome allocation is governed directly by external metabolite concentrations, rather than indirectly through an internal energy concentration variable (a). Their consumer-resource model differs in that it does not include metabolite production, meaning consideration of thermodynamic reversibility is not possible. This simpler consumer-resource model allows them to find a species' relative reaction expression reactions using a constrained maximisation procedure, rather than assuming them to be fixed as our model did (Eq 9). Using their model, they obtain conditions under which more species would survive than there are substrates in the system, something that was not observed in our model under any scenario (see Fig ST2). We suspect, that allowing species to dynamically alter their metabolic strategies means that the enhanced coexistence previously observed for specific communities (see [14, 15]) becomes accessible for a far broader range of microbial communities. However, truly reconciling their results with ours would require the development and analysis of a unified model, combining thermodynamic constraints with adaptive reallocation of proteome between reactions. The additional dependence of reaction rate on waste-product concentrations would significantly complicate the constrained optimisation procedure, making constructing this unified model challenging. However, it could yield substantial insight.

   This paper shows a clear overlap in methodology with our. We, therefore, now mention their work in the "proteome partitioning" subsection of the Model section of the main text (lines 87–88). We have also added a new SI subsection (S6.2) that discusses this work in detail.

   More generally, a comparison of assumptions and how they affect results would be very important for me to really understand what the thermodynamic framework adds.

   As mentioned in response to the previous comment a new section of the supplementary text (S9) has been added, which lays out and justifies the assumptions made in our model.

4. The authors should also dialogue more explicitly with [16] (Ref 20 main text) which also showed that the primary driver of diversity is energy availability due to the number of intermediate substrates. What new do I learn from incorporating all these additional assumptions?

   In Marsland *et al.* (2019)'s model, maximum diversity is limited *directly* by the total amount of energy leaked by species (as metabolites), which determines effectively how many substrates (metabolites) are available (in sufficient quantities) to sustain a diverse set of species. Therefore, diversity is higher when energy supply is large *with* a significant proportion of it leaked. The consumer-resource dynamics in this model involve relatively

short transients because metabolite uptake, allocation to growth and leakage is instantaneous, and any metabolite may be (probabilistically) produced. In contrast, in our model, community assembly occurs with the constraint that the initially supplied substrate can only be broken down into metabolites one or two steps below it in a free-energy hierarchy. This, combined with the embedded cellular proteome dynamics and thermodynamic constraints on free-energy availability on cellular growth rate, enforces a sequential and delayed differentiation of substrates during assembly time. This effectively introduces time lags between new substrates becoming available (see Fig 2C in the manuscript), leading to substantially longer transients during which rare species can establish on newly-generated resources (compare Fig 2A of our manuscript with Fig 1E in Marsland *et al.*). Therefore, two key new insights/results emerge from our model that would not have been possible by using the Marsland *et al.* model:

- The availability of free-energy controls the rate of community assembly and turnover (development).
- For a top-down assembly procedure (and without additional immigration events), the number of survivors is governed by the rate of niche generation (i.e. the rate of ecosystem development).

No doubt these insights come at the cost of a more complex model, but we argue that this model captures key biological details that are key to understanding microbial community assembly in nature. We have added a discussion of the Marsland *et al.* paper [16] and how its results compare to ours to a new SI subsection (S6.3). The supplementary text section discussing this (and other) work is refered to and summarised in a new paragraph in the Discussion section (lines 440–457).

It was also shown that ordinary consumer resource models can also produce modularity (see [5]). It would be helpful to distinguish the results of this new model from those in these papers.

Indeed, the modularity observed in our model systems is a consequence of the consumer-resource model itself rather than the thermodynamic constraints *per se*. In order to make clear that this result is not novel to our model, we now cite this paper suggested by the Reviewer (lines 510–511). We would also like to note that our modularity results are now displayed in a new section (S7) of the supplementary text.

5. The two-step interaction calculation is very reminiscent of a cavity calculation. Perhaps, since everything is random one can figure out average effect size by considering invasion growth rates and changes in steady-state concentrations (see for example [17]; [18]). It would be interesting to better understand the relationship between these methods and whether one is in effect calculating the cavity susceptibilities.

   We thank the reviewer for this insightful suggestion. Partly inspired by the two studies cited by the reviewer (and also [19]), we did consider ways to analytically calculate the effective interactions (susceptibilities). However, we could not obtain analytic solutions for these susceptibilities, because in contrast to [17] & [18] reaction rates in our model depend not just on substrate concentrations but also waste-product concentrations. This generates a coupling between resource dynamics (i.e. where the concentration of one metabolite can effect the rate a different metabolite is consumed at), which makes the system far harder to solve analytically. Thus, a closed form analytic solution for the steady-state concentrations as a function of the species populations cannot be found. Although the growth rates of invading species can be calculated instantaneously, in the absence of an analytic result for the susceptibilities no link between this growth rate and eventual invasion success can be made. Therefore, performing a calculation similar to the cavity calculation is not possible

without substantially simplifying assumptions. Therefore, we instead use a numerical perturbation method to calculate the instantaneous impact of each species on every other species at steady state as a measure of interactions between species. We have added an explicit justification of this choice to the start supplementary section S5, and also now briefly address this issue when introducing our novel interaction types (lines 213–218).

References

1. Noor, E. *et al.* The Protein Cost of Metabolic Fluxes: Prediction from Enzymatic Rate Laws and Cost Minimization. *PLOS Computational Biology* **12,** e1005167 (Nov. 2016).
2. Niebel, B., Leupold, S. & Heinemann, M. An upper limit on Gibbs energy dissipation governs cellular metabolism. *Nature Metabolism* **1,** 125–132 (Jan. 2019).
3. Basan, M. *et al.* Overflow metabolism in Escherichia coli results from efficient proteome allocation. *Nature* **528,** 99–104 (Dec. 2015).
4. Akbari, A., Yurkovich, J. T., Zielinski, D. C. & Palsson, B. O. The quantitative metabolome is shaped by abiotic constraints. *Nature Communications* **12,** 3178 (Dec. 2021).
5. Marsland, R., Cui, W. & Mehta, P. A minimal model for microbial biodiversity can reproduce experimentally observed ecological patterns. *Scientific Reports* **10,** 3308 (Dec. 2020).
6. Lynch, M. & Marinov, G. K. The bioenergetic costs of a gene. *Proceedings of the National Academy of Sciences* **112,** 15690–15695 (Dec. 2015).
7. Ni, B., Colin, R., Link, H., Endres, R. G. & Sourjik, V. Growth-rate dependent resource investment in bacterial motile behavior quantitatively follows potential benefit of chemotaxis. *Proceedings of the National Academy of Sciences* **117,** 595–601 (Jan. 2020).
8. Underwood, K. A., Swartz, J. R. & Puglisi, J. D. Quantitative polysome analysis identifies limitations in bacterial cell-free protein synthesis. *Biotechnology and Bioengineering* **91,** 425–435 (Aug. 2005).
9. Weiße, A. Y., Oyarzún, D. A., Danos, V. & Swain, P. S. Mechanistic links between cellular trade-offs, gene expression, and growth. *Proceedings of the National Academy of Sciences* **112,** E1038–E1047 (Mar. 2015).
10. Scott, M., Klumpp, S., Mateescu, E. M. & Hwa, T. Emergence of robust growth laws from optimal regulation of ribosome synthesis. *Molecular Systems Biology* **10,** 747–747 (Aug. 2014).
11. Scott, M., Gunderson, C. W., Mateescu, E. M., Zhang, Z. & Hwa, T. Interdependence of Cell Growth and Gene Expression: Origins and Consequences. *Science* **330,** 1099–1102 (Nov. 2010).
12. Chen, K. *et al.* Thermosensitivity of growth is determined by chaperone-mediated proteome reallocation. *Proceedings of the National Academy of Sciences* **114,** 11548–11553 (Oct. 2017).
13. Pacciani-Mori, L., Suweis, S., Maritan, A. & Giometto, A. Constrained proteome allocation affects coexistence in models of competitive microbial communities. *The ISME Journal* **15,** 1458–1477 (May 2021).
14. Posfai, A., Taillefumier, T. & Wingreen, N. S. Metabolic Trade-Offs Promote Diversity in a Model Ecosystem. eng. *Physical Review Letters* **118,** 028103 (Jan. 2017).
15. Li, Z. *et al.* Modeling microbial metabolic trade-offs in a chemostat. *PLOS Computational Biology* **16,** e1008156 (Aug. 2020).
16. Marsland, R. *et al.* Available energy fluxes drive a transition in the diversity, stability, and functional structure of microbial communities. *PLOS Computational Biology* **15,** e1006793 (Feb. 2019).
17. Advani, M., Bunin, G. & Mehta, P. Statistical physics of community ecology: a cavity solution to MacArthur's consumer resource model. *Journal of Statistical Mechanics: Theory and Experiment* **2018,** 033406 (2018).

18. Cui, W., Marsland, R. & Mehta, P. Effect of Resource Dynamics on Species Packing in Diverse Ecosystems. *Physical Review Letters* **125,** 048101 (July 2020).
19. Marsland, R., Cui, W. & Mehta, P. The Minimum Environmental Perturbation Principle: A New Perspective on Niche Theory. *The American Naturalist* **196,** 291–305 (Sept. 2020).
